# From diabetes to tumor growth: unravelling the impact of glucose-lowering therapies

**DOI:** 10.1007/s12020-026-04644-1

**Published:** 2026-05-04

**Authors:** R. Modica, A. Liccardi, V. Zamponi, A. Gagliardi, A. Nistor, G. Veroi, A. Arecco, A. Faggiano, A. Colao

**Affiliations:** 1 Endocrinology, Diabetology and Andrology Unit, Department of Clinical Medicine and Surgery, Federico II University of Naples, Naples, Italy; 2 Department of Clinical and Molecular Medicine, Sapienza University of Rome, Rome, Italy; 3 Department of Experimental Medicine, Section of Medical Physiopathology, Nutrition Science and Endocrinology, Sapienza University, Rome, Italy; 4 Department of Endocrinology, San Camillo-Forlanini Hospital, Rome, 00152 Italy; 5 Endocrinology Unit, Department of Internal Medicine and Medical Specialties, School of Medical and Pharmaceutical Sciences, University of Genova, Genova, 16132 Italy

**Keywords:** cancer, risk factor, antidiabetics drugs, tumors, diabetes

## Abstract

**Purpose:**

Diabetes mellitus and cancer are two expanding global health burdens that share upstream determinants, yet diabetes may also contribute to malignancy risk through hyperglycaemia/AGE-related stress, insulin resistance with compensatory hyperinsulinaemia, obesity-related inflammation, and tumour microenvironment modulation. This narrative review synthesizes mechanistic plausibility and critically appraises the highest-level clinical evidence on the oncologic safety signals of glucose-lowering therapies, with a focus on cancer incidence captured within randomized diabetes trials.

**Methods:**

argeted searches of PubMed/MEDLINE and Embase were complemented by manual reference screening. Mechanistic and translational data were summarized separately from clinical evidence. For each drug class, we prioritized pivotal randomized controlled trials and cardiovascular outcome trials (phase 3–4, large, practice-defining), extracting malignancy reporting collected as adverse events/serious adverse events or events of special interest. When randomized evidence was limited, high-quality meta-analyses and selected real-world studies were used for context, clearly distinguished from prespecified randomized evidence.

**Results:**

Metformin shows a predominantly neutral effect on cancer incidence, with possible indirect protective signals but no trials specifically designed to assess chemoprevention. Thiazolidinediones demonstrate an overall neutral malignancy profile, with early concerns regarding bladder cancer not confirmed in adjudicated analyses. GLP-1 receptor agonists and dual GLP-1/GIP agonists consistently show no increase in overall or site-specific cancer risk, with emerging signals of reduced obesity-related malignancies. SGLT2 inhibitors display class-wide oncologic neutrality, with heterogeneous and inconclusive drug-specific patterns in meta-analyses. Randomized evidence confirms that insulin, including long-acting analogues, does not increase cancer risk.

**Conclusion:**

Current randomized evidence supports a predominantly neutral effect of antidiabetic therapies on cancer incidence, with some agents showing promising signals of protection. This interpretation is constrained by non-prespecified endpoints, limited oncologic adjudication, short follow-up relative to tumour latency, low site-specific event counts, and potential competing-risk effects. Dedicated long-term trials and registry-linkage strategies with cancer-focused endpoints are needed to clarify whether any therapies confer true protection or warrant targeted monitoring.

## Introduction

Over the past few decades, the global public health landscape has undergone profound changes driven by the escalating prevalence of chronic noncommunicable diseases (NCDs). Among these, diabetes mellitus and cancer represent two of the most significant causes of morbidity and mortality worldwide, posing a major burden on individuals, healthcare systems, and societies. Their increasing incidence and prevalence, fueled by environmental, demographic, and behavioral determinants, have led experts to describe them as *“silent pandemics”* [1, 2]. According to the World Health Organization (WHO), NCDs account for approximately 74% of global deaths—equivalent to 41 million deaths each year—with cardiovascular diseases, cancer, chronic respiratory diseases, and diabetes being the leading contributors [1]. The global prevalence of diabetes alone has risen from 108 million in 1980 to more than 530 million in 2021, with projections indicating over 1.3 billion affected individuals by 2050 [3]. Similarly, cancer incidence continues to grow, with an estimated 20 million new cases and 9.7 million deaths recorded in 2022, and projections forecasting up to 28.4 million new cases annually by 2040 [4]. While these conditions historically dominated health statistics in high-income countries, their burden is increasingly shifting toward low- and middle-income regions. This reflects global demographic and epidemiological transitions characterized by population aging, rapid urbanization, sedentary lifestyles, and widespread exposure to shared metabolic and environmental risk factors [1]. This convergence underscores the urgency of integrated prevention strategies aimed at addressing common upstream determinants.

### Materials and methods

This manuscript is a narrative review aimed at (i) summarizing the biological mechanisms that underpin the association between diabetes mellitus and cancer and (ii) critically appraising the highest-level clinical evidence on the oncologic safety signals of glucose-lowering therapies. For the mechanistic framework, we performed a targeted literature appraisal of preclinical and translational studies addressing hyperglycaemia, hyperinsulinaemia/insulin–IGF signalling, obesity-related inflammation, oxidative stress, and tumour microenvironment modulation, including drug-class–specific pathways (metformin, thiazolidinediones, GLP-1 receptor agonists/dual GIP–GLP-1 agonists, DPP-4 inhibitors, SGLT2 inhibitors, insulin, and sulfonylureas). Literature searches were conducted in PubMed/MEDLINE and Embase, complemented by manual screening of reference lists from relevant reviews and landmark trials. For the clinical evidence section, for each drug class, we prioritized pivotal randomized controlled trials (RCT) and major cardiovascular outcome trials (CVOT) (phase 3–4, multicenter, event-driven when applicable) that were clinically practice-defining, included contemporary standard-of-care comparators, had large sample size and intermediate-to-long follow-up, and provided available safety reporting on malignant neoplasms (as adverse events/serious adverse events or events of special interest). When multiple trials were available, preference was given to those with the largest exposure time and most granular published safety data.” When RCT data were limited, recent high-quality meta-analyses were used to contextualize findings, while clearly distinguishing randomized evidence from post hoc analyses and observational studies. Given that cancer outcomes were rarely prespecified or adjudicated endpoints in diabetes trials, oncologic findings were interpreted as exploratory and assessed in light of key methodological constraints, including follow-up duration, number of events, and limited statistical power for site-specific malignancies.

### Diabetes mellitus and cancer: epidemiology and risk factors

Diabetes mellitus is a chronic metabolic disorder characterized by impaired insulin secretion, insulin action, or both, leading to persistent hyperglycemia and multisystem complications, particularly involving the vascular and nervous systems [5]. Type 2 diabetes (T2DM), accounting for over 90% of cases, is strongly linked to aging populations and modifiable lifestyle determinants. The International Diabetes Federation (IDF) 2025 Diabetes Atlas estimates that 589 million adults aged 20–79 were living with diabetes in 2024 (11.1% of the global population in that age group), with projections indicating a 45% increase by 2050 (853 million), and the largest proportional increases expected in Africa and in the Middle East and North Africa [6]. Complementary evidence from the Global Burden of Disease Study similarly highlights a sustained rise, with a substantial proportion of diabetes-related disability attributable to elevated body mass index (BMI) [3]. Major modifiable risk factors include excess adiposity—especially visceral obesity—physical inactivity, unhealthy dietary patterns, tobacco use, harmful alcohol consumption, sleep deprivation, and psychosocial stress; non-modifiable determinants include older age, family history, ethnicity, and prior gestational diabetes or polycystic ovary syndrome [5].

Cancer comprises a heterogeneous group of diseases characterized by uncontrolled cellular proliferation with potential for local invasion and distant metastasis, and remains among the leading causes of death globally [7]. The most common malignancies include lung, colorectal, breast, prostate, and gastric cancers. Demographic projections indicate a continued rise in incidence, driven largely by population aging and urbanization [4]. Cancer risk reflects the interplay of genetic susceptibility and environmental exposures, with major modifiable determinants including tobacco use, harmful alcohol consumption, unhealthy dietary patterns, excess body weight, and physical inactivity [8]. Non-modifiable factors include age, inherited genetic mutations (e.g., BRCA), family history, and comorbidities, including diabetes, which may further contribute to overall cancer risk [8].

### Epidemiological convergence

**Fig. 1:**
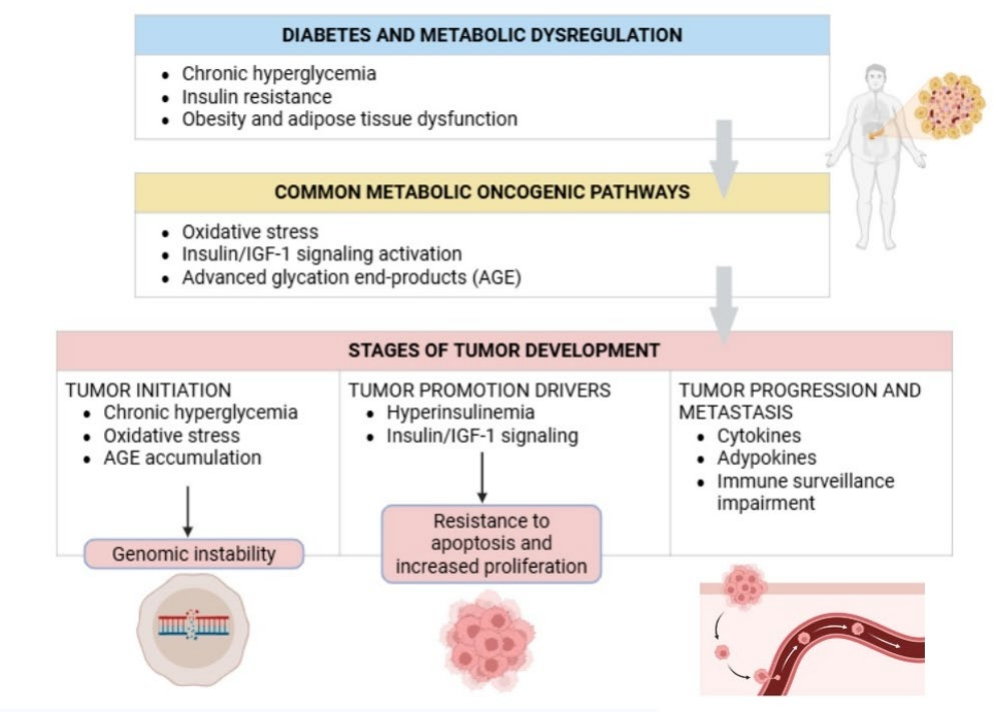
Link between diabetes mellitus type 2 and tumorigenesis

The concurrent increase in diabetes and cancer incidence is not coincidental. These conditions share multiple upstream determinants, particularly obesity, sedentary behavior, and unhealthy dietary patterns, resulting in overlapping metabolic and inflammatory milieus [1]. Beyond shared risk factors, growing epidemiological evidence suggests that diabetes itself may independently influence the risk of several malignancies, notably pancreatic, liver, colorectal, endometrial, and breast cancer, whereas an inverse association has been reported for prostate cancer [9]. Mechanistically, chronic hyperglycemia, insulin resistance with compensatory hyperinsulinemia, low-grade inflammation, and oxidative stress have been implicated as biological links between T2DM and tumorigenesis, acting through convergent pathways that may affect cellular proliferation, survival, and immune surveillance (Fig. 1.) [10–12]. This epidemiological and mechanistic convergence provides the rationale for examining whether glucose-lowering therapies are oncologically neutral, potentially protective, or associated with signals requiring monitoring, while acknowledging that most RCT were designed for cardiometabolic outcomes rather than cancer endpoints [13].

 [3, 5–9, 14–16](Cosmin Stan & Paul, 2024; Wang et al., 2020) [10–13, 17–34].

## Interplay between antidiabetic drugs and cancer Table 1

**Table 1:** Putative mechanisms of action of hypoglycemic drugs in cancer metabolism

Mechanism of action	Pathways of action	Class of drugs
Protein synthesis	AMPK/mTOR/S6K	Metformin, Thiazolidinediones, dual GIP/GLP1, GLP1-RAs, DDP-4i, SGLT2i, insulin
Inflammation	IGF-1R/PI3K/Akt/MAPK/NF-kB	Metformin, Thiazolidinediones, dual GIP/GLP1, GLP1-RAs, DDP-4i, insulin, sulfonylureas
	TNF-α, interleuchin and cytokines	Thiazolidinediones, dual GIP/GLP1, GLP1-RAs, SGLT2i, insulin
	Lipogenesis	Metformin, insulin
Cell cycle regulation	CDKs	Metformin, Thiazolidinediones, dual GIP/GLP1, GLP1-RAs
Apoptosis	Caspases	Thiazolidinediones
	Bax/Bcl-2	Dual GIP/GLP1, GLP1-RAs
Invasion and metastasis	Metalloproteasis	Thiazolidinediones, dual GIP/GLP1, GLP1-RAs, DDP-4i
	ICAM-1, VCAM-1	Dual GIP/GLP1, GLP1-RAs
Angiogenesis	SDF1/CXCL12	DPP4i
	VEGF	Insulin

Accumulating clinical and experimental data indicates that glucose-lowering therapies exert markedly divergent effects on cancer development and progression, ranging from protective to potentially harmful. The convergence of diabetic and neoplastic pathophysiology occurs through interconnected metabolic axes, including dysregulated insulin/IGF-1 signaling, chronic low-grade inflammation, oxidative stress, and aberrant cellular bioenergetics, which create both shared disease mechanisms and therapeutic opportunities. This relationship between diabetes, glucose-lowering therapies, and cancer encompasses distinct biological and clinical domains that should not be conflated. At the earliest stage, primary carcinogenesis (cancer initiation/incidence) may be influenced by chronic hyperglycaemia and advanced glycation end-products (AGEs), which can promote genomic instability, oxidative stress, and pro-oncogenic signaling [35, 36]. In parallel, insulin resistance with compensatory hyperinsulinaemia may primarily act as a tumor promoter, enhancing proliferation and survival via insulin/IGF-1 pathways once transformed clones are present. Beyond initiation and promotion, the metabolic and inflammatory microenvironment (adipokines, cytokines, immune surveillance, and oxidative stress) may shape tumor progression and metastatic potential, potentially interacting with treatment-related weight changes and systemic metabolism [37, 38]. Finally, some drug classes might exert direct tumor-cell effects in selected contexts (e.g., modulation of glucose transporters such as SGLT2, AMPK–mTOR signaling, or immune-related pathways), which could theoretically influence tumor growth, response to anticancer therapies, and cancer-specific survival in patients with established malignancy. Importantly, these domains correspond to different measurable outcomes. Most CVOT and large diabetes RCTs capture incident cancer signals as safety events, not tumor progression, treatment response, or cancer-specific survival, and they are rarely powered or designed to discriminate these endpoints [39]. Accordingly, apparent “neutrality” in incidence does not exclude potential effects on progression in specific oncologic settings, and conversely, signals from oncology-oriented repurposing studies do not directly translate into population-level cancer prevention. This conceptual framework highlights a key evidence gap: robust data remain limited for progression- and survival-focused outcomes, underscoring the need for dedicated long-term studies and oncology-specific trials when these endpoints are of interest.

In this section, we summarize mechanistic and translational evidence (primarily in vitro, animal, and early oncology-oriented clinical studies) supporting biological plausibility for drug–cancer interactions; clinical evidence on malignancy signals from pivotal diabetes trials and cardiovascular outcome trials, where cancers were generally collected as safety events, is presented separately in next section.

### Metformin

Metformin exerts pleiotropic antineoplastic effects through coordinated modulation of energy metabolism, growth-factor signaling, and oncogenic transcriptional programs.

Mechanistic evidence from in vitro and animal models supports several anticancer pathways, although these data primarily provide biological plausibility rather than direct estimates of cancer risk in humans.

One of the main pathways involving this biguanide is the AMPK (AMP-activated protein kinase) signaling pathway. The LKB1-dependent activation of AMPK leads to inhibition of mTOR (mammalian target of rapamycin), suppressing its activity and resulting in decreased phosphorylation of S6 kinase and reduced global and cap-dependent protein synthesis in breast, colon, and prostate cancer cells [40, 41]. Concurrently, AMPK phosphorylates and inhibits acetyl-CoA carboxylase (ACC), diminishing malonyl-CoA production and de novo lipogenesis; metformin reduces expression of key lipogenic enzymes, ATP citrate lyase, ACC1, and fatty acid synthase—limiting membrane biogenesis and energy storage required for tumor growth [42].

By inhibiting hepatic gluconeogenesis, metformin also decreases circulating insulin and IGF-1 levels, attenuating the IGF-1R/PI3K/Akt axis in insulin-responsive cancers [40]. In colorectal cancer cells, metformin prevents Akt-mediated β-catenin phosphorylation at Ser552 via an AMPK–PI3K–Akt pathway, sequestering β-catenin at the plasma membrane and abolishing Wnt/β-catenin–driven transcription [43, 44]. Additionally, various studies suggest that metformin promotes G1 cell-cycle arrest by downregulating cyclin D1 in breast cancer, while Sahra et al. demonstrated that metformin can also trigger p53-dependent apoptosis and G2–M arrest in prostate cancer cells [45, 46].

These intertwined mechanisms, AMPK-mediated mTORC1 inhibition, suppression of lipogenesis, reduction of mitogenic insulin/IGF-1 signaling, inhibition of β-catenin activation, cell-cycle arrest, and induction of apoptosis, establish metformin as a promising dual-purpose agent for glycemic control and chemoprevention in diverse malignancies. Early translational clinical studies in oncology settings have explored whether these mechanisms translate into antiproliferative effects in humans, typically using short-term biomarker endpoints rather than cancer incidence.

Clinically, metformin’s mechanisms translate into variable outcomes [47–50].

In a Phase IIa trial of obese, non-diabetic patients with prior colorectal adenomas, metformin did not alter rectal mucosal pS6 or Ki67 despite metabolic improvements [47]. Conversely, in a “window-of-opportunity” trial in nondiabetic women with breast cancer who were candidates for surgery, metformin reduced Ki-67 in luminal B tumors, but only in insulin-resistant women (HOMA-IR > 2.8), highlighting metabolic context as a determinant of antiproliferative efficacy [48]. Exploratory post hoc clinical evidence has also been reported in selected oncologic populations.

In a post hoc CLARINET analysis, diabetic NET patients in the placebo arm taking metformin had longer progression-free survival (85.7 vs. 38.7 weeks) than those not on metformin, whereas statistical significance was not reached in patients randomized to receive somatostatin analogues. The authors of the study suggest that metformin may provide clinical benefit in patients not receiving active antitumor therapy, while its effect becomes negligible when a treatment that inhibits the mTORC1 axis, such as lanreotide, is introduced [49].

These integrated data support metformin’s role as a dual-purpose agent—targeting metabolic drivers of tumorigenesis and directly inhibiting oncogenic pathways—warranting further phase III trials stratified by metabolic phenotype and incorporating molecular biomarkers to optimize chemopreventive and therapeutic strategies.

### Thiazolidinediones

Thiazolidinediones (TZDs) exert anticancer effects through PPAR-γ–mediated modulation of differentiation, inflammation, and cell-cycle regulation, with supporting clinical and preclinical evidence. Most of the mechanistic rationale derives from in vitro and animal models and should be interpreted as biological plausibility rather than direct evidence of cancer risk modification in humans.

TZDs (e.g., pioglitazone) activate the nuclear receptor PPAR-γ, which heterodimerizes with RXR to regulate transcription of genes controlling adipogenesis, lipid metabolism, and inflammatory responses [51]. They induce G1 arrest through coordinated modulation of cell-cycle regulators.

In in vitro studies on breast cancer cells, troglitazone increased the G1-phase fraction from 51% to 69% and decreased cyclin D1 [52]. The suppression of cell growth mediated by the reduction of cyclin D1 was also confirmed in pancreatic cancer cells in the study by Toyota et al., while other authors reported dose-dependent growth inhibition in prostate, gastric, and lung cancer cells [53–55].

In hepatocellular carcinoma cells, troglitazone promotes p27Kip1 accumulation, stabilizing this key CDK inhibitor against ubiquitin-mediated degradation and preventing cancer cell growth, and induces apoptosis through caspase-3 activation [56, 57].

PPAR-γ activation also suppresses NF-κB signaling and reduces proinflammatory cytokines (TNF-α, IL-6) and COX-2 expression, creating a less permissive tumor microenvironment.

TZD-induced PPAR-γ activation promotes adiponectin secretion, which is known to activate AMPK in colon and prostate cancer cells, further inhibiting mTORC1 signaling and protein synthesis and amplifying antiproliferative effects [41, 58].

PPAR-γ activation promotes cellular differentiation across multiple cancer types. In colon cancer, TZDs induce adipocyte-like morphological changes with increased cytoplasm-to-nucleus ratios and upregulation of differentiation markers, including carcinoembryonic antigen and cytokeratins 18 and 19 [59].

PPAR-γ activation inhibits cancer cell invasion and metastasis by transcriptionally reprogramming genes controlling cell adhesion and extracellular matrix turnover. In hepatocellular carcinoma models, rosiglitazone upregulates cell adhesion molecules while suppressing pro-metastatic factors, including matrix metalloproteinases MMP9 and MMP13. These effects translate into significant reductions in both in vitro invasiveness (60% reduction) and in vivo metastatic burden [60]. Clinical evidence cited in this section largely comes from early-phase oncology studies (i.e., trials conducted in patients with established cancer) and informs potential therapeutic repurposing rather than population-level cancer incidence in diabetes.

In a clinical setting, a phase II trial in metastatic renal cell carcinoma combining pioglitazone with anti-inflammatory agents achieved a 35% objective response rate, with 76% of patients attaining disease control [61]. In contrast, phase II trials on refractory metastatic breast cancer and chemotherapy-resistant metastatic colorectal cancer failed to demonstrate clinical benefit from TZD treatment [62, 63]. Keith et al. described a slight chemopreventive role of pioglitazone, without reaching statistical significance, in former smokers, highlighting the need to identify the highest-risk lesions to fully understand TZDs’ chemopreventive potential [64].

The fragmented clinical evidence and limited data from RCTs emphasize the need for further studies to better understand the anticancer effects of TZDs.

### GLP-1RAs and dual GIP/GLP-1

GLP-1 receptor agonists (GLP-1RAs) and dual GIP/GLP-1 receptor agonists (glucose-dependent insulinotropic polypeptide/GLP-1 receptor agonists), such as tirzepatide, influence tumor biology through multiple interconnected biochemical mechanisms. The mechanistic framework described below is primarily supported by preclinical studies and provides biological plausibility; it does not by itself establish effects on cancer incidence or clinical oncologic outcomes. These agents inhibit key oncogenic signaling pathways, including PI3K/Akt/mTOR and MAPK/ERK, leading to suppression of cancer cell proliferation via upregulation of p21Cip1/Waf1 and p27Kip1, while promoting apoptosis through modulation of Bax/Bcl-2 expression and activation of caspase-3. Additionally, AMPK activation induces a metabolic shift from anabolic to catabolic states, reversing the Warburg effect by reducing glucose uptake and glycolytic activity in tumor cells [65, 66].

Adipokine regulation, characterized by decreased leptin and increased adiponectin, further limits tumor progression, especially in obesity-related cancers [67]. GLP-1RAs also influence extracellular matrix remodeling and cell adhesion by regulating matrix metalloproteinases (MMPs), their inhibitors (TIMPs), and adhesion molecules (ICAM-1, VCAM-1), limiting tumor invasion and metastatic spread [68, 69].

Beyond direct effects on tumor cells, these drugs modulate the tumor microenvironment by suppressing proinflammatory cytokines such as IL-6 (involved in the IL-6/STAT3 pathway) and TNF-α, reducing inflammation that promotes tumor progression and metastasis [70, 71].

Emerging data indicate significant immunomodulatory activity, notably through enhancement of natural killer (NK) cell cytotoxicity mediated via the CD98-mTOR-glycolysis axis, leading to increased production of interferon-γ and granzyme B [72].

Preclinical and clinical studies demonstrate these effects across various cancers, including thyroid, pancreatic, breast, ovarian, endometrial, colorectal, hepatocellular carcinoma, and glioma.

The followed clinical evidences largely refers to translational/oncology-oriented studies or indirect clinical observations, and should not be conflated with diabetes RCT designed for cardiometabolic outcomes.

Breast cancer growth is suppressed in adipokine-rich microenvironments, and dulaglutide induces epigenetic reactivation of tumor suppressor genes [73].

Exenatide suppresses cell migration and promotes apoptosis via caspase activation in ovarian carcinoma, whereas liraglutide and tirzepatide exhibit antiproliferative effects in endometrial carcinoma [69, 74].

Hepatocellular carcinoma exhibits reduced tumor burden and enhanced immune cytotoxicity [38].

In glioma, liraglutide activates AMPK and inhibits PI3K/Akt/mTOR signaling, inducing autophagy and apoptosis [75, 76].

Potential safety concerns, including thyroid C-cell hyperplasia, originated from rodent toxicology studies in which GLP-1RAs induced C-cell changes and occasional medullary thyroid carcinoma. Importantly, these findings may have limited translatability because rodent thyroid C-cells show higher GLP-1 receptor expression and a different calcitonin biology than humans. Accordingly, in humans no consistent signal for medullary thyroid carcinoma has been demonstrated in large randomized programs to date, although continued pharmacovigilance is warranted [77, 78].

### DPP-4 inhibitors

Dipeptidyl peptidase-4 inhibitors (DPP-4i) modulate tumor biology through both enzymatic and non-enzymatic mechanisms, influencing proliferation, apoptosis, immune surveillance, and the tumor microenvironment.

These mechanistic observations are primarily derived from preclinical research and should be interpreted as hypothesis-generating.

By inhibiting DPP-4/CD26 enzymatic activity, these drugs increase circulating levels of bioactive incretins (GLP-1, GIP) and other peptides, indirectly affecting cancer-relevant signaling pathways such as PI3K/Akt, MAPK/ERK, and AMPK, thereby suppressing tumor growth and promoting apoptosis [79, 80].

Non-enzymatic functions of DPP-4/CD26 involve interaction with extracellular matrix components and immune cell receptors, modulating T-cell activation, chemotaxis, and natural killer (NK) cell cytotoxicity, which can enhance antitumor immune responses [81].

Preclinical models indicate that DPP-4 inhibition can reduce metastasis and tumor invasiveness. For example, in pancreatic and colorectal cancer models, sitagliptin decreased cell migration and invasion by downregulating MMP2/MMP9 and inhibiting epithelial-to-mesenchymal transition (EMT) markers [82, 83].

Conversely, in some contexts, DPP-4 inhibition may enhance tumor growth by increasing stromal-derived factor-1 (SDF-1/CXCL12) signaling, which promotes angiogenesis and tumor cell homing, highlighting the dual and context-dependent effects of these drugs [84, 85].

Clinically, observational studies suggest a neutral to modestly protective effect on cancer incidence, though RCT data are limited. Meta-analyses indicate no increased overall cancer risk with DPP-4i, while effects may differ by cancer type and patient metabolic status [86–88]-.

### SGLT2 inhibitors

Sodium-glucose cotransporter 2 inhibitors (SGLT2i) affect cancer biology primarily through systemic metabolic modulation and direct effects on tumor cell metabolism. Recent comprehensive reviews have highlighted the rationale for repurposing SGLT2i in oncology, based on tumor glucose dependence and evidence that SGLT transporters may be expressed in selected malignancies (e.g., lung adenocarcinoma, pancreatic, renal, prostate and breast cancers) [89]. By inducing glycosuria, SGLT2i reduce circulating glucose and insulin levels, thereby diminishing insulin/IGF-1–driven proliferative signaling in insulin-sensitive tumors [90]. In addition, systemic effects such as weight reduction and shifts in the inflammatory and oxidative milieu have been proposed as indirect mechanisms that could influence the tumor microenvironment [89].

In vitro studies demonstrate that SGLT2 is expressed in multiple cancer types, including pancreatic, renal, and prostate cancers, and SGLT2i can directly inhibit glucose uptake in these tumor cells, leading to energy stress, AMPK activation, and inhibition of mTORC1 signaling, ultimately resulting in cell-cycle arrest and apoptosis [91, 92]. However, translational interpretation requires caution because several in vitro studies employ suprapharmacologic drug concentrations; pharmacokinetic considerations suggest that clinically achievable free concentrations may be substantially lower, raising the possibility of off-target effects in some experimental settings [89].

Additional mechanisms include suppression of epithelial-to-mesenchymal transition, reduction of reactive oxygen species, and modulation of the tumor microenvironment via decreased inflammatory cytokines [93].

Preclinical data show reduced tumor growth and metastasis in xenograft models of lung, breast, and pancreatic cancer treated with canagliflozin or empagliflozin [94, 95]. Beyond direct tumor effects, SGLT2i have also been hypothesized to interact with anticancer therapies—particularly by mitigating cardiotoxicity and potentially enhancing antitumor activity in preclinical models—although these observations remain preliminary and not validated in oncology RCT [96].

Clinical evidence remains limited, with observational studies showing no significant increase in cancer incidence and ongoing trials investigating potential antitumor benefits. A recent narrative review concluded that available meta-analyses of randomized trials do not show a significant increase in overall malignancy risk, while early concerns regarding bladder cancer signals appear inconsistent and may vary by agent and context, warranting continued surveillance rather than firm causal inference [96].

### Insulin

Insulin plays a significant role in cancer progression by activating overlapping signaling pathways that control cell growth, metabolism, and survival. Central to this process is the activation of the insulin receptor, which triggers downstream pathways such as the mechanistic target of rapamycin (mTOR) pathway, providing essential substrates to tumor cells. At the same time, insulin stimulates the insulin receptor substrate (IRS)/phosphoinositide 3-kinase (PI3K)/Akt pathway and the mitogen-activated protein kinase (MAPK) cascade. Both pathways regulate cell cycle progression, inhibit apoptosis, and promote angiogenesis [97–99].

These pathways have been implicated in enhancing proliferation and survival across multiple cancer types, including glioblastoma, thyroid cancer, lung cancer, hepatocellular carcinoma, and colorectal cancer. In colorectal cancer, for example, insulin upregulates acetyl-CoA acetyltransferase 1 (ACAT1), a key enzyme for lipid metabolism and mitochondrial function, supporting tumor growth and metastasis [100–104].

Hyperinsulinemia, often associated with obesity and insulin resistance, amplifies these effects by disturbing the balance of angiogenic regulators in the tumor microenvironment. This results in increased production of pro-angiogenic factors, such as vascular endothelial growth factor (VEGF) and leptin, while reducing the anti-angiogenic molecule adiponectin. It also contributes to chronic low-grade inflammation, characterized by elevated levels of inflammatory cytokines, including interleukin-6 (IL-6) and tumor necrosis factor-alpha (TNF-α), further facilitating tumor progression [105, 106].

Moreover, due to the structural similarity between insulin and insulin-like growth factor 1 (IGF-1), insulin can bind to and activate the IGF-1 receptor, enhancing mitogenic and anti-apoptotic signaling pathways that promote cellular proliferation and survival [107–109].

### Sulfonylureas

Sulfonylureas primarily act by stimulating pancreatic beta-cells to secrete insulin, which can lead to hyperinsulinemia and potentially promote tumor growth through enhanced mitogenic signaling, including IGF-1. Epidemiological studies suggest that sulfonylurea use may be associated with an increased risk of certain cancers, such as colorectal and pancreatic [110, 111].

However, preclinical studies indicate that some sulfonylureas, particularly glibenclamide, may exert antineoplastic effects. These include inducing cell cycle arrest and inhibiting tumor proliferation through modulation of ATP-sensitive potassium channels [112]. Sulfonylureas may also influence the tumor microenvironment (TME) by preventing activation of cancer-associated fibroblasts, affecting metabolic reprogramming, and enhancing immune responses against tumors [113].

Other sulfonylureas, such as chlorpropamide, gliclazide, glipizide, and acetohexamide, have similarly demonstrated anti-tumor effects by reducing pro-survival cytokines, angiogenesis, invasion, and metastasis [114, 115]. The complex and sometimes opposing effects of sulfonylureas underscore the need for further mechanistic studies to clarify their role in cancer progression and potential therapeutic applications. Unfortunately, robust RCT cancer data lacking.

## Evidence from clinical trial (Table 2)

**Table 2:** Randomized Controlled Trials Reporting Cancer-Related Outcomes

Drug Class	Trial	Intervention / Comparator	Population (*N*)	Follow-up	Cancer Outcome Handling	Key Cancer Results	Overall Signal
**Metformin**	UKPDS 34 (Lancet (London, England), 1998)	Metformin vs. conventional therapy	753	10,7 yrs	Cancer deaths recorded	NS	Neutral
	UKPDS 80 (Holman et al., 2008)	Metformin vs. conventional therapy vs. conventional therapy+diet	4,209	17 yrs	Cancer as cause of death	NS Cancer is the second cause of death	Neutral
	DPP (Knowler et al., 2002)	Metformin vs. placebo vs. lifestyle in IGT population	3,234	2,8 yrs	Not reported	No data	Not assessable
	HOME (Kooy et al., 2009)	Metformin+insulin vs. insulin	390	4.3 yrs	Not reported	No data	Not assessable
**TZDs**	PROactive (Dormandy et al., 2005)	Pioglitazone vs. placebo	5,238	~ 3 yrs	Cancer as SAE	NS	Neutral
	DREAM (DREAM Trial Investigators et al., 2006)	Rosiglitazone vs. placebo in IFG and/or IGT population	5,269	3 yrs	Not reported	No data	Not assessable
	ADOPT (Kahn et al., 2006)	Rosiglitazone vs. metformin vs. glyburide	4,360	4 yrs	Cancer as SAE	No signals published	Neutral
	**RECORD** **(Home et al.**,** 2009)**	**Rosiglitazone add-on vs. control**	**4**,**447**	**5**,**5 yrs**	**Cancer as prespecified endpoint**	**NS**	**Neutral**
**GLP-1RAs**	**LEADER** (Marso, Daniels, et al., 2016)	**Liraglutide vs. placebo**	**9**,**340**	**3**,**8 yrs**	**Cancer as prespecified endpoint**	**NS**	**Neutral**
	SUSTAIN-6 (Nauck and Quast, 2021)	Semaglutide vs. placebo	3,297	~ 2 yrs	Cancer as SAE	NS	Neutral
	PIONEER 6 (Husain et al., 2019)	Oral semaglutide vs. placebo	3183	15,9 m	Cancer as SAE	NS	Neutral
	EXSCEL (Holman et al., 2017)	Exenatide vs. placebo	14,716	3,2 yrs	Pancreas/thyroid prespecified	NS	Neutral
	REWIND (Gerstein et al., 2019)	Dulaglutide vs. placebo	9,901	5,4 yrs	Cancer as SAE	NS	Neutral
**DPP-4is**	SAVOR-TIMI 53 (Leiter et al., 2016)	Saxagliptin vs. placebo	16,492	2,1 yrs	Cancer as SAE	NS	Neutral
	TECOS (Buse et al., 2017)	Sitaglipin vs. placebo	14,671	3 yrs	Cancer as SAE	NS	Neutral
	CARMELINA (Rosenstock et al., 2019)	Linagliptin vs. placebo	6,979	2,2 yrs	Cancer as SAE	NS	Neutral
**SGLT2is**	EMPA-REG (Zinman et al., 2015)	Empagliflozin vs. placebo	7,020	3.1 yrs	Not reported	No data	Not assessable
	CANVAS (Neal et al., 2017)	Canagliflozin vs. placebo	10,142	~ 4 yrs	Cancer as SAE	NS	Neutral
	DECLARE-TIMI 58 (Wiviott et al., 2019)	Dapagliflozin vs. placebo	17,160	~ 4 yrs	Cancer as SAE	NS	Neutral
	VERTIS CV (Cannon et al., 2020)	Ertugliflozin vs. placebo	8,246	~ 3 yrs	Not reported	No data	Not assessable
**Insulin**	**ORIGIN** **(ORIGIN Trial investigators**,** 2012)**	**Glargine vs. standard**	**12**,**537**	**6.2 yrs**	**Cancer as prespecified endpoint**	**NS**	**Neutral**
	4-T Study (Holman, Sourij and Califf, 2014)	Biphasic/prandial/basal insulin	708	3 yrs	Cancer as SAE	NS	Neutral
	DEVOTE (Marso, McGuire, et al., 2016)	Degludec vs. glargine	7,637	~ 2 yrs	Cancer as SAE	NS	Neutral

TZDs: Thiazolidinediones; GLP-1Ras: GLP-1 receptor agonists; DPP-4is: Dipeptidyl peptidase-4 inhibitors; SGLT2is: Sodium-glucose cotransporter 2 inhibitors; SAE: serious adverse event; NS: no significant

Clinical evidence on glucose-lowering therapies and cancer arises from two conceptually distinct domains. First, ‘population-level’ evidence from diabetes RCTs and CVOT evaluates cancer incidence as a safety outcome in patients treated primarily for cardiometabolic indications; these trials are generally not designed or powered for oncologic endpoints. Second, ‘drug repurposing’ studies in oncology test glucose-lowering agents as adjunct anticancer therapies in patients with established malignancy, focusing on cancer-specific outcomes such as response, progression-free survival, or overall survival. These domains address different questions: cancer incidence versus tumor behavior/progression, and should not be interpreted interchangeably.

### Metformin

Evidence from diabetes RCToverall suggests a neutral to possibly protective association between metformin and malignancy risk, although none of the pivotal trials were designed with cancer incidence as a primary or secondary endpoint.

In UKPDS 34, 753 overweight patients with newly diagnosed type 2 diabetes were randomized to intensive therapy with metformin or to conventional treatment [116]. Over a median follow-up of 10.7 years, metformin reduced all-cause mortality by 36% and diabetes-related endpoints by 32%. Cancer was not a prespecified endpoint, but cause-specific mortality was adjudicated, and cancer-related deaths were numerically lower in the metformin arm (13 vs. 21; 3.5 vs. 4.9 per 1,000 patient-years; RR 0.71, 95% CI 0.29–1.76), without reaching statistical significance. When metformin was combined with sulfonylureas, overall mortality increased (14 vs. 6; 9.0 vs. 3.7 per 1,000 patient-years; RR 2.47, 95% CI 0.70–8.66), but no cancer-specific excess was observed.

In the extended post-trial follow-up, which provided a median of 17 years of observation, sustained cardiovascular benefits of early intensive therapy were confirmed [117]. Cancer was recorded as a cause of death and accounted for 24.2% of deaths—second only to cardiovascular causes—but no formal, prespecified analysis of incident malignancies was conducted, and no differences in cancer mortality emerged between treatment groups.

The Diabetes Prevention Program (DPP) randomized 3,234 individuals with impaired glucose tolerance to placebo, intensive lifestyle intervention, or metformin (850 mg twice daily) [118]. Over 2.8 years, metformin reduced incident diabetes by 31% versus placebo. However, cancer outcomes were neither prespecified nor reported, and the original publication provides no data on malignancies, precluding conclusions on short-term oncologic effects. Similarly, in the HOME trial, which evaluated the addition of metformin to insulin therapy, serious adverse events were systematically collected, but malignancies were not reported as a distinct safety category, suggesting either very low event numbers or lack of systematic oncologic analysis [119].

Beyond diabetes RCTs, metformin has also been tested directly in oncology settings, these findings belong to the ‘drug repurposing’ domain. In the STAMPEDE platform, a large randomized phase 3 trial evaluated the addition of metformin (850 mg twice daily) to standard of care in non-diabetic patients with metastatic hormone-sensitive prostate cancer starting androgen deprivation therapy; metformin did not significantly improve overall survival in the overall population (HR 0.91, 95% CI 0.80–1.03; *p* = 0.15), while improving adverse metabolic effects associated with androgen deprivation therapy [120]. Additional evidence comes from real-world observational analyses. Shi et al. (2026) used a population-based “sequential target trial emulation” approach, meaning that electronic health record data were analyzed by explicitly specifying the design of an ideal RCT (eligibility, treatment initiation, time-zero, follow-up, and outcomes) and then emulating that trial month-by-month in the observational dataset to reduce common biases (e.g., immortal time and selection bias). In this study of men with prostate cancer receiving androgen deprivation therapy, metformin monotherapy was not associated with delayed hormone therapy failure, although it was associated with improved overall survival; as non-randomized evidence, these findings remain hypothesis-generating and may be influenced by residual confounding [121]. Overall, RCT of metformin support a neutral association with cancer risk, with no signal of harm and a possible, though unproven, indirect protective effect reflected in reduced all-cause and cardiovascular mortality in UKPDS. The absence of trials with cancer as a prespecified endpoint, limited adjudication of incident malignancies, and insufficient statistical power underscore the need for dedicated RCT to clarify metformin’s potential chemopreventive role.

### Thiazolidinediones

RCT of TZDs were primarily designed to evaluate cardiovascular and glycemic outcomes rather than malignancy. In most studies, cancers were captured as serious adverse events (SAEs) or prespecified safety outcomes, but not as primary endpoints.

The PROactive trial randomized 5,238 patients with type 2 diabetes and established macrovascular disease to pioglitazone or placebo [122]. Malignant neoplasms were prespecified as SAEs and systematically recorded. Overall, the incidence of malignant tumors was balanced between groups (97 vs. 99 patients). An apparent excess of bladder cancer was observed in the pioglitazone arm (14 vs. 6; *p* = 0.069), while breast cancer was less frequent (3 vs. 11; *p* = 0.034). After blinded re-adjudication excluding lesions likely present before randomization, the imbalance in bladder cancer diminished (6 vs. 3; *p* = 0.309). Although non-significant, this transient observation fuelled concerns about pioglitazone-associated bladder cancer risk that later studies attempted to address.

By contrast, the DREAM trial evaluated rosiglitazone versus placebo in 5,269 adults with impaired fasting glucose or impaired glucose tolerance to assess diabetes prevention over a median of 3 years. Cancer incidence was neither prespecified nor reported, and malignancies do not appear in the primary or supplementary publications, so DREAM does not contribute direct evidence on oncologic risk [123].

The ADOPT trial randomized 4,360 patients with newly diagnosed type 2 diabetes to rosiglitazone, metformin, or glyburide, with durability of glycemic control as the primary endpoint [124]. In the trial protocol, cancer was explicitly included in the definition of SAEs, requiring mandatory reporting for any fatal, life-threatening, or cancer-related event. However, the main publication does not provide malignancy data, implying that no clinically relevant oncologic signal emerged during follow-up or that events were too few to warrant dedicated analysis.

This evidence gap was partially addressed in RECORD, which randomized 4,447 patients already on metformin or sulfonylurea to addition of rosiglitazone versus the alternative agent [125]. In this trial, malignant neoplasms were prespecified safety outcomes and adjudicated by an independent committee over a mean follow-up of 5.5 years. Overall cancer incidence did not differ significantly between groups (5.7% with rosiglitazone vs. 6.6% with active control; *p* = 0.20). Site-specific analyses showed no excess of prostate (1.3% vs. 1.8%), breast (1.0% vs. 1.6%), colon (0.5% vs. 0.6%), or bladder cancers (0.3% vs. 0.2%) in the rosiglitazone arm. Pancreatic cancer occurred less frequently with rosiglitazone (< 0.1% vs. 0.6%; *p* = 0.0074), a finding interpreted as likely due to chance, given the very small number of events and limited biological plausibility. A subsequent pooled re-analysis of ADOPT and RECORD extracted all malignancy events across nearly 39,000 patient-years [126]. Cancer incidence was comparable among rosiglitazone, metformin, and glyburide (1.12, 1.03, and 1.31 cases per 100 patient-years, respectively), and hazard ratios between treatments were close to unity, confirming the neutral oncologic profile suggested by RECORD.

In conclusion, randomized evidence indicates that TZDs have a largely neutral profile with respect to cancer risk. The transient bladder cancer imbalance observed in PROactive did not persist in subsequent trials or pooled analyses, and comprehensive post hoc evaluations have not identified a consistent association between TZD exposure and increased overall or site-specific malignancy.

### GLP-1 receptor agonists and dual GLP-1/GIP agonists

GLP-1RAs are now central in the management of type 2 diabetes and obesity, delivering robust glycemic control, clinically meaningful weight loss, and cardiovascular and renal protection. Dual GLP-1/GIP agonists such as tirzepatide have further expanded this therapeutic class. Safety concerns regarding pancreatic cancer and medullary thyroid carcinoma originated from rodent C-cell findings and early pharmacovigilance signals. In the major cardiovascular and phase 3 programs, neoplasms were systematically captured as safety outcomes, but none of the trials were primarily designed or powered to assess cancer risk, and cancer endpoints were generally exploratory or post hoc.

In LEADER, 9,340 patients with type 2 diabetes and high cardiovascular risk were randomized to liraglutide or placebo, with a median follow-up of 3.8 years [127]. Liraglutide significantly reduced major adverse cardiovascular events (HR 0.87; 95% CI 0.78–0.97). A dedicated analysis of malignant neoplasms, based on prespecified safety data, showed similar rates of total malignant tumors (liraglutide 1.06 vs. placebo 1.12; HR not significant) and no clear increase in pancreatic cancer, although numerically more pancreatic events were noted. LEADER was not primarily designed to assess neoplasm risk, but its data exclude any major increase in overall malignancy with liraglutide. Calcitonin levels remained stable over time, and no cases of medullary thyroid carcinoma or C-cell hyperplasia were reported [127].

In SUSTAIN-6, subcutaneous semaglutide reduced cardiovascular risk in 3,297 high-risk patients over ~ 2 years [127, 128]. Malignancies were recorded as safety outcomes and did not differ between semaglutide and placebo, including pancreatic cancer. Across the broader SUSTAIN and PIONEER phase 3 programs, only a small number of thyroid cancers were reported, without a consistent dose–response pattern; in PIONEER 6, oral semaglutide did not increase malignant neoplasms versus placebo during approximately 16 months of follow-up [129].

The EXSCEL trial, which randomized 14,752 patients to once-weekly exenatide or placebo, similarly monitored malignancies as safety events and reported no excess in pancreatic cancer (15 vs. 16 cases) or thyroid carcinoma in the exenatide arm [130]. In REWIND, dulaglutide versus placebo in 9,901 individuals followed for a median of 5.4 years showed neutral cancer outcomes overall, with a slight numerical increase in “thyroid neoplasms” but no cases of medullary thyroid carcinoma [131].

For dual GLP-1/GIP agonists, the evidence base is smaller but reassuring. A meta-analysis of 13 tirzepatide trials (13,761 participants; 26–72 weeks of follow-up) found no increase in overall cancer (RR 0.78; 95% CI 0.53–1.16) or in specific tumor types [132]. Although tirzepatide induces dose-dependent increases in calcitonin, no medullary or papillary thyroid carcinoma has been reported in controlled trials, and observational datasets focusing on thyroid cancer show no increased risk, with some signals of protection against obesity-related cancers [78, 132].

In summary, data from CVOT, program-wide safety analyses, meta-analyses, and observational cohorts support a predominantly neutral effect of GLP-1 RAs and dual GLP-1/GIP agonists on cancer risk in the short-to-intermediate term. Emerging signals suggest possible protection against obesity-associated cancers such as colorectal, endometrial, and ovarian malignancies, likely mediated by weight loss and broader metabolic improvements. At the same time, small numerical imbalances in rare thyroid tumors and heterogeneous real-world findings justify continued pharmacovigilance and, ideally, dedicated long-term trials with oncologic endpoints as these agents are increasingly used in younger, lower-risk populations.

### DPP-4 inhibitors

DPP-4i have been extensively evaluated in CVOT, in which malignancies were prospectively collected as adverse events of special interest, although none of these trials was designed or powered to assess cancer incidence as a primary endpoint. Consequently, oncologic findings should be interpreted as exploratory and hypothesis-generating.

In the SAVOR-TIMI 53 trial, 16,492 patients with type 2 diabetes and high cardiovascular risk were randomized to saxagliptin or placebo and followed for a median of 2.1 years [133]. At least one malignancy was reported in 326 patients (3.8%) receiving saxagliptin and 362 patients (4.3%) receiving placebo (HR 0.89; 95% CI 0.77–1.04). Site-specific analyses showed broadly balanced distributions across cancer types. Pancreatic cancer occurred in 5 patients (0.07%) in the saxagliptin group and 12 patients (0.15%) in the placebo group.

In TECOS, 14,671 patients with established cardiovascular disease were randomized to sitagliptin or placebo [134]. Pancreatic cancer occurred in 9 patients (0.042%) in the sitagliptin group and 14 patients (0.066%) in the placebo group (HR 0.66; 95% CI 0.28–1.51). No statistically significant difference was observed.

In CARMELINA, 6,979 high-risk patients were randomized to linagliptin or placebo and followed for a median of 2.2 years [135]. All cancers were reported in 116 patients (3.3%) receiving linagliptin and 134 patients (3.8%) receiving placebo. Adjudication-confirmed pancreatic cancer occurred in 11 patients (0.3%) in the linagliptin group and 4 patients (0.1%) in the placebo group. Only one case per group was deemed possibly related to study drug exposure by independent oncology experts.

Available randomized evidence does not demonstrate an increased overall cancer risk with DPP-4i, although limited follow-up and low event rates preclude definitive conclusions regarding rare or long-latency malignancies.

### SGLT2 Inhibitors

SGLT2i have transformed the management of type 2 diabetes through improvements in glycemic control, weight, and cardiovascular and renal outcomes. Early development, however, raised concern about potential cancer risk—particularly bladder and breast cancer—based on numerical imbalances in pooled safety data with dapagliflozin. In all CVOT, malignancies were systematically captured as adverse events or events of special interest but were not primary or secondary efficacy endpoints, and trials were not powered for oncologic outcomes.

In EMPA-REG, 7,020 patients with type 2 diabetes and established cardiovascular disease were randomized to empagliflozin or placebo. Empagliflozin significantly reduced the primary composite endpoint of cardiovascular death, nonfatal myocardial infarction, or nonfatal stroke (HR 0.86; 95% CI 0.74–0.99) [136]. Malignant neoplasms were collected as safety outcomes, but the primary publication does not provide a detailed breakdown of cancer incidence by treatment arm. No major cancer safety signal was reported, yet the absence of granular data and lack of oncologic power limit definitive conclusions on cancer risk.

The CANVAS Program, integrating CANVAS and CANVAS-R and including 10,142 participants, evaluated canagliflozin versus placebo in high-risk patients [137]. Canagliflozin reduced the primary composite cardiovascular outcome (HR 0.86; 95% CI 0.75–0.97) and slowed progression of albuminuria. Malignancies were collected as adverse events; the incidence of cancer was nearly identical (3.5% vs. 3.4%), and no specific cancer signal emerged.

In DECLARE–TIMI 58, 17,160 patients with type 2 diabetes and established atherosclerotic cardiovascular disease or multiple risk factors were randomized to dapagliflozin or placebo. Predefined “events of special interest” included malignancie [138]. Dapagliflozin significantly reduced hospitalization for heart failure (HR 0.73; 95% CI 0.61–0.88) and improved renal outcomes, while overall cancer incidence remained similar between groups (3.9% vs 4.1%), with no statistically significant increase in bladder or breast cancer despite prior safety concerns.

The VERTIS CV trial included 8,246 patients with type 2 diabetes and atherosclerotic cardiovascular disease, randomized to ertugliflozin or placebo [139]. The trial demonstrated non-inferiority for major adverse cardiovascular events. Malignant neoplasms were collected within the overall safety database, but cancer incidence was not a primary or dedicated secondary endpoint, and the published article does not provide a detailed incidence of malignancy by treatment arm. Thus, although no major cancer signal was reported, oncologic conclusions from VERTIS CV must be considered exploratory.

These CVOT provide consistent, robust evidence that SGLT2i do not meaningfully increase cancer risk over intermediate follow-up (≈ 2.5–4 years). While malignancies were not primary endpoints and event numbers are relatively low, the convergence of data across multiple agents (empagliflozin, canagliflozin, dapagliflozin, ertugliflozin) and tumor sites is reassuring.

Beyond individual CVOT, several meta-analyses and real-world studies have explored cancer risk more systematically. A network meta-analysis of 46 RCTs found no significant increase in overall cancer incidence (RR 1.08; 95% CI 0.96–1.21), but suggested potential drug-specific signals: a higher risk of bladder cancer with SGLT2i, particularly empagliflozin (OR 4.49; 95% CI 1.21–16.73), and a possible protective effect of canagliflozin against gastrointestinal cancers (OR 0.15; 95% CI 0.04–0.60) [140]. Dicembrini et al. subsequently pooled 27 RCTs (> 48,000 participants) and reported no significant increase in overall malignancies with SGLT2i(MH-OR 0.98; 95% CI 0.77–1.24), nor elevated risk for specific cancers, including bladder, breast, prostate, hepatic, pancreatic, or gastrointestinal tumors [141].

A larger meta-analysis by Shi et al. synthesized 77 RCTs (~ 89,000 individuals) and again showed no class-wide increase in malignancy risk, but pointed to heterogeneous, drug-specific patterns: ertugliflozin and dapagliflozin appeared to increase overall cancer risk in some comparisons, whereas empagliflozin was associated with reduced malignancy versus active comparators but a possible excess of digestive tract tumors versus placebo [142]. These inconsistencies, coupled with relatively short trial durations and low event counts, highlight the challenge of defining subtle anticancer or pro-oncogenic effects from metabolic trials.

Real-world data introduce additional nuance. Observational cohorts, including a Taiwanese-based meta-analysis of > 400,000 participants, reported no significant increase in breast cancer risk or cancer-related mortality with SGLT2i compared with DPP-4i, but suggested a substantially reduced risk of hepatocellular carcinoma (HR ≈ 0.54; 95% CI 0.42–0.69), possibly linked to metabolic and anti-inflammatory benefits and supported by emerging preclinical data.

Overall, current evidence supports a predominantly neutral class effect of SGLT2i on cancer incidence, with possible drug- and site-specific heterogeneity that remains to be fully elucidated. The lack of cancer-focused endpoints, limited follow-up, and low event rates argue strongly for long-term observational surveillance and dedicated oncologic trials in populations treated chronically with SGLT2i.

### Insulin

Insulin remains a cornerstone therapy for type 1 diabetes and advanced type 2 diabetes. Concerns about its oncologic safety arise from its mitogenic and anti-apoptotic properties via insulin and IGF-1 receptor activation. These concerns apply particularly to long-acting analogues such as glargine and degludec. Several large RCTs and observational studies have therefore examined whether exogenous insulin influences cancer incidence and mortality, with varying degrees of prespecification for oncologic outcomes.

The ORIGIN trial was the first large RCT explicitly designed to evaluate the long-term impact of insulin glargine on both cardiovascular events and cancer [143]. It enrolled 12,537 adults with impaired fasting glucose, impaired glucose tolerance, or early type 2 diabetes, randomized to once-daily insulin glargine (titrated to fasting glucose ≤ 95 mg/dL) versus standard care. Over a median follow-up of 6.2 years, the incidence of new cancers—a prespecified safety endpoint—was 8.7% in the glargine group and 9.1% in the standard care group (HR 0.99; 95% CI 0.88–1.11). Cancer-related mortality was similarly neutral (HR 0.94; 95% CI 0.77–1.15), and no differences emerged for specific cancer types. Among randomized studies, ORIGIN provides the most robust long-term evidence on the oncologic safety of basal insulin glargine. Over a median follow-up of 6.2 years, incident cancers and cancer-related mortality were comparable between glargine and standard care, supporting the absence of a clinically meaningful signal of harm. Nevertheless, as with most diabetes RCTs, site-specific malignancy analyses remain limited by low event counts and should be interpreted as exploratory rather than definitive.

The 4-T Study randomized 708 patients with type 2 diabetes inadequately controlled on oral therapy to biphasic, prandial, or basal insulin regimens over 3 years [144]. The trial focused on glycemic control and hypoglycemia; malignancies were captured within serious adverse events but not prespecified or reported as a dedicated outcome, and no regimen was associated with a discernible excess of cancer diagnoses in subsequent analyses.

In DEVOTE, 7,637 high-cardiovascular-risk patients with type 2 diabetes were randomized to insulin degludec versus insulin glargine U100 and followed for a mean of nearly 2 years [145]. The primary endpoint was cardiovascular, but neoplasms were collected as part of the safety assessment. The incidence of malignancies was comparable between degludec and glargine, with no statistically significant differences.

By contrast, observational studies have produced more heterogeneous results. Early analyses of large insurance and registry databases suggested a possible dose-dependent association between insulin glargine and cancer—particularly breast cancer but these findings were highly sensitive to statistical adjustment and lacked detailed information on key confounders such as obesity, diabetes duration, and concurrent therapies [146, 147]. When RCT are evaluated collectively, the apparent association disappears. A meta-analysis of 16 RCTs reported no significant increase in overall cancer incidence among insulin-treated patients (relative risk 1.06; 95% CI 0.93–1.20) [32]. Taken together, the most robust evidence indicates that exogenous insulin, including long-acting analogues such as glargine and degludec, does not increase the risk of developing cancer or cancer-related mortality. Nevertheless, given the biological plausibility of mitogenic effects, especially in individuals requiring very high cumulative doses, continued pharmacovigilance remains prudent, particularly in patients with severe insulin resistance.

## Strengths and limitations of CVOT-derived cancer data

Most human evidence on cancer outcomes with glucose-lowering therapies derives from CVOT and other large randomized diabetes trials in which malignancies are generally collected as safety events rather than prespecified efficacy endpoints. This has important implications for interpretation. First, follow-up is often limited relative to the latency of many solid tumors and, despite large sample sizes, the absolute number of cancer events, particularly for site-specific malignancies, remains low, resulting in limited statistical power to detect modest risk differences. Second, competing-risk and survivor effects may shape observed cancer incidence when therapies reduce cardiovascular mortality and extend time at risk for cancer detection, potentially complicating comparisons across treatment arms. Third, post hoc and exploratory analyses are inherently constrained by multiplicity and by variable granularity in safety reporting and event classification across trials. Finally, observational studies can complement trial data by offering longer follow-up and broader populations, but remain vulnerable to confounding by indication and differential screening intensity. CVOT-derived cancer findings are best interpreted as safety signals requiring contextualization rather than definitive evidence of neutrality or protection.

## Future directions: toward a more definitive assessment of cancer risk

While current evidence derived from CVOT and observational studies provides valuable signals, a more definitive assessment of cancer risk will require dedicated and methodologically refined research strategies. A key priority is long-term RCT in which cancer outcomes are incorporated as primary or co-primary endpoints. Compared with CVOT, such trials should include formal power calculations for overall and site-specific malignancies, systematic adjudication of oncologic events, and follow-up durations sufficient to capture the latency of solid tumor development. These design features would also facilitate clearer separation of effects on cancer incidence from those on progression, recurrence, and cancer-specific survival. In parallel, large-scale registry-based linkage studies represent a complementary and highly valuable approach. Integrating clinical trial cohorts, diabetes registries, or administrative healthcare databases with national cancer registries would enable long-term surveillance in real-world settings, substantially increasing statistical power for rare and site-specific cancers and improving generalizability to more heterogeneous populations, including individuals often underrepresented in RCT (e.g., patients with prior malignancy). Finally, future investigations should move toward biomarker-stratified and phenotype-driven designs. The oncologic impact of glucose-lowering therapies may not be uniform but could vary according to underlying metabolic and hormonal milieus; stratification based on hyperinsulinemia, insulin resistance, IGF-1 activity, inflammatory markers, or visceral adiposity may help identify subgroups with differential susceptibility and provide mechanistic insight linking epidemiologic associations to biologically plausible pathways.

## Conclusions and final remarks

Mechanistic and translational evidence supports biologically plausible links between diabetes-related metabolic dysfunction and cancer biology, including pathways driven by hyperglycaemia/AGE-related stress, hyperinsulinaemia–insulin/IGF signalling, obesity-associated inflammation, and tumor microenvironment modulation. These mechanisms may operate differently along the cancer continuum, so outcomes from oncology-oriented repurposing studies should not be conflated with population-level cancer incidence signals reported in diabetes trials. On balance, the available randomized evidence suggests that most glucose-lowering drug classes have a broadly reassuring oncologic safety profile in the short-to-intermediate term, with occasional drug- or site-specific observations that justify continued pharmacovigilance. Therefore, current treatment choices should remain guided primarily by established cardiometabolic benefits and patient-centered considerations, while acknowledging that long-latency and site-specific cancer outcomes remain incompletely characterized.

## Data Availability

All data generated or analyzed during this study are included in this article. Further inquiries can be directed to the corresponding author.
